# How to Rest the Mind

**Published:** 1870-11

**Authors:** W. H. Lathrop

**Affiliations:** Prof. of Physiology and Psychology, Detroit Medical College


### Selections.

How to Rest the Mind.—By W. H. Lathrop, M. D., Prof. of Physiology and Psychology, Detroit Medical College.—Said the dying Anquetil to his physician, "You see a man *dying full of life*." A New York banker of vigorous physical appearance recently went to Europe for his health. Though to all external appearance perfectly well, he felt that his mind had been overworked and needed rest. Soon after his return home from Europe, however, he had an attack of apoplexy, occasioned, it seems probable, by a mistake with regard to the *kind* of diversion which he needed. He had exchanged one form of excitement for another, like a man who should take the blower down from his grate and substitute another of a different pattern in its place. It would seem much more advisable in such a case to go into the country, where the mind could be rested by an easy, quiet, *drifting* life. Mr. Greeley's cabbage garden might not have been a bad investment, after all, even though that attractive vegetable was raised, as he tells us, at an expense of thirteen dollars a head. Agriculture, digging in the dirt, seems to be almost a specific for an overwrought brain.

Dean Swift predicted, as the sequel proved, that he "should die first at the top," for he felt that he was wearing out his brain too fast. It is said that "people is most happy whose annals are tiresome," and doubtless the same is true to some extent with regard to individuals; that life is not always, by any means, the most happy or the most useful that is filled with excitement.

Dr. Carpenter expresses very clearly the principles governing the wear and tear of brain: "Like all other tissues actively concerned in the vital operations, nervous matter is subject to a waste or disintegration, which bears an exact proportion to the activity of its operations. Every idea, every emotion, every act of volition, and every perception, however passive or fleeting, is necessarily attended by a waste and decay of a certain portion of the brain tissue." So intimate is this association of mind and matter, that Prof.

Huxley says: "Matter may be regarded as a form of thought, and thought may be regarded as a property of matter."

In view, therefore, of this constant waste, some attention must be paid to repair, and the method of securing this repair of the mind must be especially adapted to the individual case. We speak of the relaxation of the mind as we do of the relaxation of a muscle; but the latter is a simple organ which may be rested merely by inactivity. The mind, on the other hand, from its complexity, does not respond to such simple treatment. Each individual mind must be studied by itself, and its proper kind of rest selected.

One of the first questions to be asked in regard to mental recruiting is, whether *quiet* or *excitement* is needed. An old lady said that some people, in looking forward to another world, were very much interested in the *occupations* of Heaven, but for her part she wanted *rest*. Physicians of large practice need this kind of recreation—the relief of having nothing particular to do for awhile.

In some young ladies' seminaries every portion of the twenty-four hours has some special duty assigned to it. The scholar feels that she is constantly in the harness. To many minds, undoubtedly, this excessive "avarice of time" is injurious. It is very refreshing for a short time every day, and for a few consecutive weeks in the year, to feel that we have no duties awaiting us.

In most cases the taste of the individual will dictate rightly whether quietness or excitement is best for him, but not always. The taste may become morbid, and in cases of insipient insanity extremely so. A gentleman in this State, who has been for many years a successful business man, has recently been so constantly engrossed in business that he is now on the verge of insanity. His extravagant bargains and recently irritable temper give certain evidences of approaching mental disease. Still his inclinations lead him to press on still further in the same mad course. Hugh Miller's case was somewhat similar. He felt an irresistible desire to make still greater mental exertions, although he knew perfectly well that such a course would injure him. These are exaggerated cases of morbid taste; but the same evil, less fully developed, is not uncommon. How often it happens that when exercise in the open air is just what the people need to strengthen them, it is the last thing which they desire to do.

There are many pleasure seekers who throng the watering places in search of excitement, when they are already dwarfed in body and mind by their devotion to that species of pleasure. Still they crave it, as a child is always ready to eat more candy. A lady who passes the greater part of the year in the fashionable circles of city life, will be most benefitted in the summer by going where the constraints of society may be laid aside. On the other hand, those whose ordinary lives are a monotonous routine of work and care, will be really benefitted by a round of excitement and active amusement. If the vacation is a long one, there should certainly be some other object in view than mere enjoyment. This applies to school children and to grown people as well. To be enjoyed, their time must be well occupied.

> "Want of occupation is not rest.
> A mind quite vacant is a mind distressed."

I have heard it remarked that to enjoy a tour in a foreign country one should select some special field of study and observation. In this way there will always be some object in view to give piquancy to the trip. A man with such a purpose will see all the lesser objects of interest quite as well while pursuing his specialty. It is said that if you want to see a star at night that is a little dim, it is best to look on one side of it. So in seeking rest for the mind, it will not do to aim at it too directly. I have often thought that such a man as Humboldt or Agassiz must receive much more enjoyment from traveling than those who travel for pleasure.

We may derive many useful suggestions for the management of the comparatively healthy mind by studying the insane. There are two points suggested in this way that seem worthy of mention. In providing a suitable atmosphere for the diseased mind, the physician pays special attention to *occupation* and *companionship*. Chambers says, that "in all the concerns of organic, as of social life, anything is preferable to *stagnation*." This condition is fatal to mental health. The mind must be employed, and the asylum endeavors to furnish the patient with some kind of amusement or employment suited to his capacity.

Perhaps no problem is more perplexing in the treatment of the insane than this. Still, in most cases, the patient can, in some way, be amused. Providing suitable companionship for the insane is quite as important, and hence the classification of the patients which is carefully made. Many patients are made delirious, and some become maniacal, from the officious, inquisitive people who crowd about their bedsides. If the physician prohibits visitors from seeing the patient, he is thought oftentimes to be unnecessarily strict. People do not realize the great harm that is done to the sick by the presence of uncongenial visitors. The case is the same with those who, though not sick, are sufficiently unwell to need recreation. A large and interesting party may be entirely spoiled by one obnoxious member, and all the anticipated recreation frustrated. Mothers who go to places of amusement with crying babies must feel this truth; those sitting near them certainly do.

Discrimination on the part of patients themselves with regard to recreation is hardly to be expected, but a physician's advice on the subject, it would seem, would certainly be heeded. Hence the subject is not unworthy of a physician's attention. Serious mental disease may be often prevented in this way. Insanity is becoming so prevalent in our country that all the causes leading to it should receive the careful study of general practitioners. A large proportion of the cases would never receive the sad diagnosis, "insanity," if their mental condition had received the early attention of their physicians.

The problem how to rest the mind, is full of importance in its application to children. Practitioners of medicine are constantly reminded that our school system, as at present constituted, is attended with many evils. The children are kept still too long. They are expected to study six hours or more in a day, when grown men find it difficult to do as much, though fully interested in their subjects of study, and gifted with mature minds. It is believed by a large body of the medical profession that the ultimate education of youth would be as efficient, both to the individuals themselves and to society, if the children did not study more than half as long.

A bright little girl, with a pale face and undefinable pains all over her body, presents herself to her physician for treatment. She has been at school since she was six years old, and she is now eleven. Her mother informs him that her child has been little inclined to take any exercise for two or three years past, though previously robust and healthy. She can not go out and play after coming from school, because she is tired or has lessons to get. Every little while she has a short illness. She is fond of school and study, is flattered by her teacher, and makes good progress in her lessons. Such a child should leave school at once, and remain away until she is well, notwithstanding the protest of the teacher that she knows more about the child than the doctor does, and it is a shame to take away her best scholar. Not only are weak and delicate children made invalids by this excessive confinement, but those who are strong evidently suffer a loss of stamina.

Now the great difficulty in reducing the number of hours of school is, that the *parents* are unwilling to have it done. They want the children out of the way. For this reason they send them to school at the earliest possible age, and wish them to remain there as many hours in the day as possible. Calisthenics and walking exercises are now quite generally introduced into school rooms, and every effort is made on the part of the teachers to secure the good health of the pupils. It is the public who need to be aroused to the importance of this subject.

If, then, it be true that a much greater length of time is spent in the schools than is required for mental discipline, could the surplus time be better utilized than in attempts to keep the children alive? If three hours a day will secure the good results that are now accomplished by six, the three hours thus gained might well be devoted to some *manual* employment that will at the same time give the children exercise and teach them useful trades. At all events, it would show them what manual labor is, and would make them feel that it is honorable. But the kid-gloved parents raise their hands in holy horror. It cannot be. They wish their sons to be *gentlemen* and their daughters to be *ladies.* So the question is settled. It is so much the fashion to hold up the professions to youthful aspiration, that any move that should make the trades seem more respectable would certainly be a great advance in our system of popular education. Better far that a mechanic should have merely the rudiments of an education, than that he should have been led to despise the honorable employment by which he makes an honest living.

To combine instruction of this utilitarian character with the ordinary curriculum of study, would hardly be practicable in our city public schools, without an entire revolution in their character. The experiment could, however, be tried in private institutions, or even in public schools in small places, where the number of children is small, and the difficulties and expenses of the experiment would be slight. This is by no means a new idea. This method of education is now in full and successful operation in reformatory institutions throughout the country. The school-ship "Massachusetts," at Boston, and a similar one at New York, are set apart for this method of education—the object being to train boys to be good sailors, while at the same time they are instructed in a sensible English education. Agricultural colleges are organized on this plan, and some military schools also may be placed in this category. The boys in these institutions are cheerful and happy, because they are well. There can be but little doubt that the best results would follow a similar course of instruction in schools throughout the country.

